# Triple and quadruple mutation of RGD motif using CRISPR-Cas9 in *him-4* locus of *Caenorhabditis elegans*

**DOI:** 10.17912/micropub.biology.000249

**Published:** 2020-05-10

**Authors:** Aileen Park, Zhongqiang Qiu, Myeongwoo Lee, Kendall Lewis, Margaret Cross, Olivia Baur, Sophia Brice, Lauren Whiteley

**Affiliations:** 1 Baylor University Department of Biology

**Figure 1:**
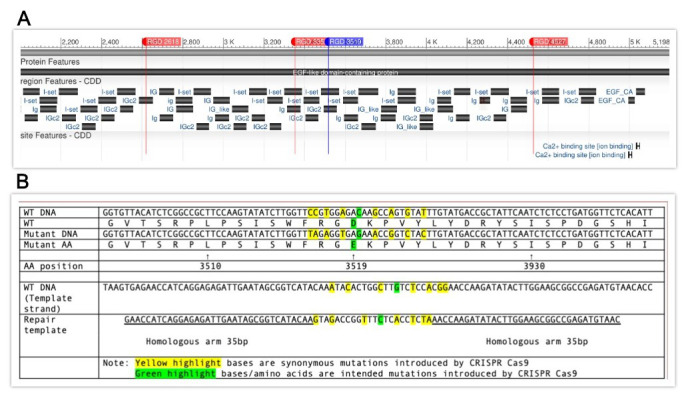
**A**. CRISPR-Cas9 was used to generate two novel alleles of the *him-4* gene. The three RGD sites in the gene that were mutated in the triple mutation are indicated in red (2618, 3352, and 4527). The fourth site that was added to create the quadruple mutation is indicated in blue (3519). Two additional motifs, 717 and 3428, are not shown here. This figure was generated using NCBI Blast. **B**. Comparison of *him-4* amino acid no. 3519 locus (wild type) and the mutant sequence. Repair template sequence and synonymous mutation schemes are also presented here. All sequences are in the 5’ to 3’ direction. *WT: wild-type. **AA: amino acid.

## Description

The *him-4* gene in *Caenorhabditis elegans* is involved in important developmental processes in the organism including basement membrane attachment, anchor cell invasion, defective cytokinesis, and gonad positioning along the basement membrane (Morrissey *et al.*, 2014). The human ortholog to *him-4*, HMCN 1 (hemicentin-1), is implicated in age related macular degeneration 1 (Thompson *et al.*, 2007). HIM-4/HMCN1 is a highly conserved extracellular matrix structural component and contains six RGD (Arg-Gly-Asp) cell-binding motifs within its 48 immunoglobin (Ig)-like repeats, while HMCN1 contains only one RGD motif (Vogel and Hedgecock, 2001). Two novel mutant alleles of *C. elegans* *him-4* were generated using CRISPR-Cas9 gene editing in this study. In one mutant allele, three of the aforementioned six RGD motifs were mutated to RGE (Arg-Gly-Glu) (Takahashi *et al.*, 2007). Four of the six RGD motifs were altered to RGE in the second mutant line. Neither of these mutant lines exhibited outstanding abnormal phenotypes when screened for behavioral or morphological abnormalities. We examined the mutants for Him (high incidence in male) phenotype, *kq8207* (0% male, n=888) and *kq8297* (0.1% male, n=963). However, the examined animals failed to show an outstanding number of males compared to N2 (0%, n=582). Mutants were also examined for behavioral abnormalities in a thrashing assay. A Mann-Whitney U Test revealed possible differences between *kq8207* mutants and N2 worms (p=0.037), and revealed no significant difference between *kq8297* mutants and N2 worms (p=0.447). 53 triple RGD mutants (average 19.85 thrashes), 51 RGD quadruple mutants (average 21 thrashes), and 51 N2 worms (average 22.16 thrashes) were used in this assay.Morrissey *et al.* demonstrated that PAT-3/INA-1integrin is essential for assembly of HIM-4/hemicentin puncta during anchor cell invasion (Morrissey *et al.*, 2014). However, integrin and HIM-4 failed to colocalize in other tissues (Vogel and Hedgecock 2001; Morrissey *et al.*, 2014). Although anchor cell invasion was not examined in this study, these *him-4* RGD mutants hold potential for further research pertaining to the functions of cell to extracellular matrix binding domains in *C. elegans*.

## Methods

In order to induce specific RGD to RGE mutations in *him-4,* CRISPR target sites were identified within the gene using the CRISPR guide RNA Selection Tool (http://genome.sfu.ca/crispr/). The triple mutation line, BU8207 (*him-4(kq8207))*, contains RGE mutations at amino acid numbers 2618, 3352, and 4527 (Figure A), in which the mutations were added sequentially into each position. The quadruple mutation line, BU8297 (*him-4(kq8297))*, contained these three loci as well as amino acid number 3519 as described here. Briefly, the mixture of custom template DNA (Temp-4HIM4RGE3519, Figure B), custom crRNA (HIM4RGD3519), tracrRNA (cat. #1072532), and Alt-R Cas9 nuclease (cat. #1081058) was annealed at room temperature (Paix *et al.*, 2015) and micro-injected into the syncytial gonad arms of N2 wild-type worms (P0) (Mello *et al.*, 1991). The mixture also included *dpy-10* crRNA as a co-CRISPR marker (Arribere *et al.*, 2014). The F1 generation was then screened for the intended mutation by identifying animals exhibiting the Dpy phenotype and by PCR genotyping using mutation specific primers (HIM4RGE3519R and HIM4RGE3519SEQF). PCR was then conducted on F2 offspring using both wild-type *him-4* and mutant-specific primers (HIM4RGD3519WTR and HIM4RGE3519SEQF) in order to isolate homozygous mutants. Once homozygotes were isolated, PCR products were sequenced in order to confirm that the mutation was successful (Psomagen Inc, Rockville, MD). The repair DNA (as well as all other oligos used to create these mutant lines) for these loci was designed and produced at IDT Inc., Coralville, IA. Both lines of mutant animals were backcrossed (1x) to N2 then studied for phenotype characterization. For Him phenotypes, N2, *kq8207,* and *kq8297* worms were self-fertilized, and males were randomly identified from NGM agar plates. The thrashing assay was performed by counting the number of body bends for 15 seconds in a 10 μl drop of M9 buffer (Lee *et al.*, 2005). A Mann-Whitney U Test was then run to confirm statistical significance of results.

crRNA sequence

HIM4RGD3519 AAUACACUGGCUUGTCUCCA

(dpy-10) ZQDP10A GCUACCAUAGGCACCACGAG

PCR Primers

HIM4RGE3519SEQF TACGCCGCAGAAGTGATTGG

HIM4RGE3519SEQR TCCTGCTTCGTTGGATGCAC

HIM4RGD3519WTR ATACACTGGCTTGTCTCCACGG

HIM4RGE3519R ACAAGTAGACCGGTTTCTCACCTCTA

Repair oligo, crRNA, and primer sequences for screening are readily available upon request.

## Reagents

BU8207 *him-4(kq8207)* and BU8297 *him-4(kq8297)* are available upon request.
